# Paclitaxel Enhances the Innate Immunity by Promoting NLRP3 Inflammasome Activation in Macrophages

**DOI:** 10.3389/fimmu.2019.00072

**Published:** 2019-01-29

**Authors:** Qiong-zhen Zeng, Fan Yang, Chen-guang Li, Li-hui Xu, Xian-hui He, Feng-yi Mai, Chen-ying Zeng, Cheng-cheng Zhang, Qing-bing Zha, Dong-yun Ouyang

**Affiliations:** ^1^Department of Immunobiology, College of Life Science and Technology, Jinan University, Guangzhou, China; ^2^Department of Cell Biology, College of Life Science and Technology, Jinan University, Guangzhou, China; ^3^Department of Fetal Medicine, the First Affiliated Hospital of Jinan University, Guangzhou, China

**Keywords:** paclitaxel, NLRP3 inflammasome, α-tubulin acetylation, MEC-17, SIRT2, epothilone B

## Abstract

Microtubules play critical roles in regulating the activation of NLRP3 inflammasome and microtubule-destabilizing agents such as colchicine have been shown to suppress the activation of this inflammasome. However, it remains largely unknown whether paclitaxel, a microtubule-stabilizing agent being used in cancer therapy, has any influences on NLRP3 inflammasome activation. Here we showed that paclitaxel pre-treatment greatly enhanced ATP- or nigericin-induced NLRP3 inflammasome activation as indicated by increased release of cleaved caspase-1 and mature IL-1β, enhanced formation of ASC speck, and increased gasdermin D cleavage and pyroptosis. Paclitaxel time- and dose-dependently induced α-tubulin acetylation in LPS-primed murine and human macrophages and further increased ATP- or nigericin-induced α-tubulin acetylation. Such increased α-tubulin acetylation was significantly suppressed either by resveratrol or NAD^+^ (coenzyme required for deacetylase activity of SIRT2), or by genetic knockdown of *MEC-17* (gene encoding α-tubulin acetyltransferase 1). Concurrently, the paclitaxel-mediated enhancement of NLRP3 inflammasome activation was significantly suppressed by resveratrol, NAD^+^, or *MEC-17* knockdown, indicating the involvement of paclitaxel-induced α-tubulin acetylation in the augmentation of NLRP3 inflammasome activation. Similar to paclitaxel, epothilone B that is another microtubule-stabilizing agent also induced α-tubulin acetylation and increased NLRP3 inflammasome activation in macrophages in response to ATP treatment. Consistent with the *in vitro* results, intraperitoneal administration of paclitaxel significantly increased serum IL-1β levels, reduced bacterial burden, dampened infiltration of inflammatory cells in the liver, and improved animal survival in a mouse model of bacterial infection. Collectively, our data indicate that paclitaxel potentiated NLRP3 inflammasome activation by inducing α-tubulin acetylation and thereby conferred enhanced antibacterial innate responses, suggesting its potential application against pathogenic infections beyond its use as a chemotherapeutic agent.

## Introduction

Paclitaxel is a first-line chemotherapeutic medicine. In clinic, it is used for the treatment of a broad spectrum of cancers, including breast cancer, lung cancer, as well as ovarian, cervical and pancreatic cancers (1–4). It is clinically used intravenously, and its distribution throughout the body is rapid, with large volumes of distribution (5). It has been known that paclitaxel is a microtubule-stabilizing agent. Mechanistically, paclitaxel binds to the β-unit of microtubule (β-tubulin), thus stabilizing the α/β polymer, and suppressing the organization capacity of centrosomes (5, 6). In mitotic cells, paclitaxel prevents the mitotic spindle from disassembly (7). Therefore, the mitotic cells treated with paclitaxel cannot proceed into metaphase and are doomed to apoptosis due to cell cycle arrest (8, 9) and reduced mitochondrial membrane potential (10). However, it has been demonstrated that paclitaxel affects microtubule dynamics at concentrations much lower than those inhibiting mitosis and cell division (11).

NLRP3 [NOD-like receptor (NLR) family, pyrin containing domain 3] is a critical cytosolic receptor that can sense bacterial, fungal and viral infections, as well as other signal molecules such as extracellular ATP (released during bacterial infection or tissue damage), nigericin (a microbial toxin derived from *Streptomyces hygroscopicus*), and monosodium urate crystals (MSU, causative factor of gout) (12). Full activation of NLRP3 inflammasome requires two inflammatory signals. The first (priming signal) is provided by interaction of a microbe-associated molecular pattern (MAMP) with its pattern recognition receptor (PRR), which induces activation of the NF-κB signaling pathway and expression of NLRP3, pro-interleukin (IL)-1β and pro-IL-18, and the second comes from various stimulators including damage associated molecular patterns (DAMPs), such as extracellular ATP (13). Upon these inflammatory stimulations, NLRP3 molecules recruit the adaptor protein ASC (apoptosis-associated speck-like protein containing a CARD) to form a large platform (i.e., NLRP3 inflammasome) for pro-caspase-1 binding, leading to its activation by autocatalytic processing. The active caspase-1 in turn proteolytically cleaves pro-IL-1β and pro-IL-18 into mature IL-1β and IL-18, which are subsequently released to potentiate the innate immunity or inflammation (14). Concomitantly, active caspase-1 also cleaves gasdermin D (GSDMD) to produce an N-terminal fragment (GSDMD-NT), which forms pores on the plasma membrane and thereby mediating programmed cell death named pyroptosis (15). Some studies have indicated that cell membrane rupture and pyroptosis is required for the release of IL-1β and other inflammatory factors (16), suggesting that pyroptosis is an important process in mediating inflammation.

Recently, published studies showed that microtubules have important roles in regulating the assembly of NLRP3 inflammasome (17, 18). It has been shown that colchicine, a microtubule-destabilizing drug that binds to β-tubulin and inhibits microtubule polymerization (19), suppresses NLRP3 inflammasome activation (17). Owing to the effect of colchicine on suppressing MSU-induced NLRP3 inflammasome activation, thus dampening IL-1β release and neutrophil recruitment (20), it has long been used in clinic for the treatment of gout (21–23). Opposite to the action mechanism of colchicine, paclitaxel stabilizes microtubule and mitotic spindle by binding to β-tubulin (24). Although paclitaxel has been implicated in NLRP3 inflammasome activation (25), its action on the inflammasome activation and the underlying mechanism are still incompletely understood. In this study, we revealed that paclitaxel dose- and time-dependently enhanced α-tubulin acetylation in lipopolysaccharide (LPS)-primed macrophages. Paclitaxel treatment greatly enhanced NLRP3 inflammasome activation, as indicated by increased mature IL-1β release, ASC speck formation and pyroptosis, in LPS-primed macrophages in response to ATP or nigericin stimulation. Inhibition of α-tubulin acetylation with resveratrol and NAD^+^, two activators of deacetylase SIRT2, or knockdown of the α-tubulin acetyltransferase MEC-17 expression attenuated paclitaxel-mediated augmentation of NLRP3 inflammasome activation. Moreover, intraperitoneal paclitaxel administration improved the survival of mice against bacterial infection. Our results suggest that paclitaxel enhanced the innate immune response against bacterial infection by enhancing NLRP3 inflammasome activation through inducing α-tubulin acetylation.

## Materials and Methods

### Reagents and Antibodies

Paclitaxel (P106868) was purchased from Aladdin (Shanghai, China), dissolved in dimethyl sulfoxide (DMSO) at 50 mM and stored at −20°C. Resveratrol (R5010), NAD^+^ (β-nicotinamide adenine dinucleotide hydrate) (N7004), ATP (A6419), lipopolysaccharide (LPS) (*Escherichia coli* O111:B4) (L4391), Hoechst 33342 (B2261), propidium iodide (PI) (P4170), anti-γ-tubulin (T5326), CF647-conjugated anti-mouse IgG (H+L), highly cross-adsorbed (SAB4600183), PMA (S1819), DMSO (D8418) and Tween-20 (P1379) were bought from Sigma-Aldrich (St. Louis, MO, USA). Nigericin (tlrl-nig), Pam3CSK4 (tlrl-pms), Poly(dA:dT) (tlrl-patn), and FLA-PA Ultrapure (purified flagellin from *P. aeruginosa*) (tlrl-pafla) were obtained from InvivoGen (San Diego, CA, USA). Epothilone B (S1364) was purchased from Selleck Chemicals (Houston, TX, USA), dissolved in DMSO at 5 mM and stored at −20°C. Dulbecco's Modifed Eagle's Medium (DMEM) medium with high glucose, Opti-MEM, fetal bovine serum (FBS), streptomycin and penicillin, Lipofectamine 2000 (11668-030), and Lipofectamine RNAiMAX (13778-075) were products of ThermoFisher/Invitrogen/Gibco (Carlsbad, CA, USA). FuGENE HD transfection reagent (E2311) was from Promega (Madison, WI, USA). The anti-NLRP3 antibody (AG-20B-0014) was purchased from Adipogen AG (Liestal, Switzerland). The antibody against actin (sc-1616-R) was purchased from Santa Cruz Biotechnology (Dallas, TX, USA). Specific antibodies against IL-1β (#12242), ASC (#67824), ASC-AlexaFluor488 (#17507), α-tubulin (#3873), acetyl-α-tubulin (#5335), horse-radish peroxidase (HRP)-linked horse anti-mouse IgG (#7076) and horse-radish peroxidase (HRP)-linked goat anti-rabbit IgG (#7074) were purchased from Cell Signaling Technology (Danvers, MA, USA). The antibodies against pro-caspase1+p10+p12 (ab179515), GSDMD (ab209845) and MEC-17 (ab58742) were purchased from Abcam (Cambridge, UK). CF568-conjugated goat-anti-rabbit IgG (H+L), highly cross-adsorbed (20103) and CF488A-conjugated goat-anti-mouse IgG, highly cross-adsorbed (20018) were obtained from Biotium (Hayward, CA, USA).

### Animals

C57BL/6 mice (6–8 weeks of age) were obtained from the Experimental Animal Center of Southern Medical University (Guangzhou, China). All the mice were acclimatized for 1 week before experiment.

### Mouse J774A.1 Macrophages

Mouse J774A.1 macrophage cell line was purchased from the Kunming Cell Bank of Type Culture Collection, Chinese Academy of Sciences (Kunming, China). Cells were cultured in DMEM supplemented with 10% FBS, 100 U/ml penicillin, 100 μg/ml streptomycin and 2 mM L-glutamine (complete DMEM medium) at 37°C in a humidified incubator of 5% CO_2_ and sub-cultured every 2–3 days by using a cell scraper to detach cells.

### Bone Marrow-Derived Macrophages (BMDMs)

Mouse BMDMs were isolated and differentiated as reported previously (26, 27). In brief, C57BL/6 mice were sacrificed and bone marrow cells in hind femora and tibias were flushed out with 10 ml of sterile PBS and collected by centrifugation at 300 × g for 5 min at 4°C. Then the cells were re-suspended in BM-Mac medium (80% DMEM medium containing 10% FBS plus 20% M-CSF-conditioned medium from L929 cells) and differentiated for 6 days at 37°C in a humidified incubator of 5% CO_2_. BMDMs were cultured in 24-well plates at 1.5 × 10 ^5^ cells/well (0.5 ml) or in 6-well plates at 1.2 × 10 ^6^ cells/well (2 ml) or in glass-bottomed dishes at 1 × 10 ^5^ cells/dish (1.5 ml) with complete DMEM medium at 37°C overnight, and were ready for experiments.

### THP-1 Cell Culture and Differentiation

THP-1 cells (ATCC) were maintained in RPMI-1640 supplemented with 10% FBS and 50 μM 2-mercaptoethanol at 37°C in a humidified incubator of 5% CO_2_. They were differentiated into macrophages by incubation with 500 nM PMA for 16 h, and then were ready for experiments.

### Western Blot Analysis

Western blotting was performed essentially as previously described (26). Briefly, total proteins were separated by sodium dodecylsulfate-polyacrylamide gel electrophoresis (SDS-PAGE) and electro-transferred to PVDF membranes (03010040001; Roche Diagnostics GmbH, Mannheim, Germany). The membranes were blocked by blocking buffer (PBS containing 3% FBS) for 1 h and incubated with indicated primary antibody overnight at 4°C, followed by incubation with HRP-linked secondary antibody. Bands were revealed with an enhanced chemiluminescence kit (BeyoECL Plus; Beyotime, Shanghai, China) and recorded by X-ray films (Carestream, Xiamen, China). The blot images were captured by FluorChem8000 imaging system (AlphaInnotech, San Leandro, CA, USA). The gray values were analyzed by AlphaEaseFC 4.0 software (AlphaInnotech).

### Precipitation of Soluble Proteins in Supernatants

Soluble protein secreted into culture supernatants (equal volume for each sample) was precipitated as previously described (26, 27). After washing 3 times with cold acetone, the precipitated proteins were re-dissolved in equal volume of 2 × SDS-PAGE loading buffer, and then secreted mature IL-1β and caspase-1p10 were analyzed by western blotting.

### Detection of Soluble IL-1β

Soluble IL-1β in culture supernatants and serum were determined by cytometric bead array (CBA) mouse IL-1β Flex Set (#560232) with the buffer (#558266) or human inflammatory cytokine kit (#551811), respectively, (BD Biosciences, San Jose, CA, USA) according to the manufacturer's instructions. Data were acquired on a flow cytometer (Attune NxT acoustic focusing cytometer; Thermo Fisher Scientific, Carlsbad, CA, USA) and analyzed by using the Attune NxT software (Thermo Fisher Scientific).

### Immunofluorescence Microscopy

Immunofluorescence analysis was performed as previously described (26). In brief, macrophages were seeded in glass-bottomed dishes and cultured at 37°C overnight. Cells were fixed in 4% paraformaldehyde for 15 min, and permeabilized with 2 ml cold methanol (−20°C) for 10 min, and then by incubated with primary antibodies at 4°C overnight, followed by staining with CF568-conjugated goat-anti-rabbit IgG and CF488A-conjugated goat-anti-mouse IgG. In separate experiments for simultaneously staining of ASC, acetylated α-tubulin and γ-tubulin, cells were incubated with rabbit anti-acetylated α-tubulin and mouse-anti-γ-tubulin, and then stained with CF647-conjugated anti-mouse IgG and CF568-conjugated goat-anti-rabbit IgG, followed by incubation with ASC-AlexaFluor488. The nuclei were revealed by Hoechst33342 (5 μg/ml) staining. Cells were observed under a Zeiss Axio Observer D1 microscope with a Zeiss LD Plan-Neofluar 40 × /0.6 Korr M27 objective lens (Carl Zeiss MicroImaging GmbH, Göttingen, Germany). Fluorescence images were captured by a Zeiss AxioCam MR R3 cooled CCD camera controlled with ZEN software (Carl Zeiss).

### Cell Death Assay

Cell death was measured by propidium iodide (PI) incorporation as described previously (26). Briefly, macrophages were cultured in 24-well plates and primed in Opti-MEM with 500 ng/ml LPS for 4 h. Then the cells were treated with indicated concentration of paclitaxel for 1 h followed by stimulation with ATP (2 or 3 mM) or nigericin (5 or 20 μM) for indicated time periods. The nuclei were revealed by Hoechst 33342 (5 μg/ml) staining. Dying cells revealed by PI (2 μg/ml) staining at room temperature for 10 min and observed immediately by live imaging using Zeiss Axio Observer D1 microscope equipped with a Zeiss LD Plan-Neofluar 20 × /0.4 Korr M27 objective lens (Carl Zeiss MicroImaging GmbH, Göttingen, Germany). Fluorescence images were captured with a Zeiss AxioCam MR R3 cooled CCD camera controlled with ZEN software (Carl Zeiss).

### Small Interfering RNA (siRNA)

The siRNA (5′-GGA TAC AAG AAG CTC TTT G-3′) duplexes targeting mouse *MEC-17* (17) and negative control (NC) siRNA were synthesized by RiboBio (Guangzhou, China). Knockdown of *MEC-17* was performed using Lipofectamine RNAiMAX according to the instructions provided by the supplier. Briefly, BMDMs were cultured in 6-well plates at 37°C overnight. The NC siRNA and *MEC-17* siRNA was added to corresponding well at a final concentration of 100 nM. Cells were cultured in DMEM medium containing 10% FBS for 72 h, and used for experiments.

### Bacterial Infection

Mouse model of bacterial infection was established as previously described (26, 28). In brief, *E. coli* DH5α was cultured and proliferated in Lysogeny broth (LB) medium at 37°C overnight, and then re-inoculated into fresh LB media and grown for 4 h at 37°C. The viable bacteria were collected by centrifugation at 2,600 × g for 10 min, washed with PBS, and then re-suspended in appropriate volume of PBS. Bacterial density was measured by using an ultraviolet-visible spectrophotometer (NanoDrop2000, Thermo Scientific) and the corresponding colony-forming units (CFUs) were determined on LB agar plates (29). Then the viable bacteria were re-suspended in PBS at 4 × 10^9^ CFU/ml. C57BL/6 mice were acclimated for a week, randomly divided into three groups and intraperitoneally injected with paclitaxel solution (5 or 10 mg/kg body weight) or vehicle (PBS). One hour later, viable *E. coli* cells (2 × 10^9^ CFU/mouse) in 0.5 ml of PBS were injected into the peritoneal cavity of each mouse. Mouse survival was monitored every 6 h for 5 consecutive days. In a paralleled experiment, mice were intraperitoneally injected with paclitaxel solution similarly. One hour later, viable *E. coli* cells (1 × 10^9^ CFU/mouse) in 0.5 ml of PBS were injected into the peritoneal cavity of each mouse and those mice were sacrificed at 8 h post bacterial infection. Their sera were collected for detection of IL-1β by CBA.

### Histopathological Analysis

Infected mice were sacrificed and the livers were isolated and fixed in 4% neutral formaldehyde, and the liver sections were stained with hematoxylin and eosin (H&E). Images were captured by the Zeiss Axio Observer D1 microscope armed with a color CCD (Zeiss Axio Observer D1).

### Statistical Analysis

Experiments were performed three times independently. Data were expressed as mean ± standard deviation (SD). Statistical analysis was performed using GraphPad Prism7.0 (GraphPad Software Inc., San Diego, CA, United States). One-way analysis of variance (ANOVA) followed by Turkey *post hoc* test and unpaired Student's *t*-test were used to analyze the statistical significance among multiple groups and between two groups, respectively. If the data were not normally distributed, Friedman (among multiple groups) and Mann-Whitney U (between two groups) were used, respectively. Kaplan–Meier survival curves were used for analysis of mouse survival and the significance was evaluated by the log-rank (Mantel–Cox) test. *P* < 0.05 was considered statistically significant.

## Results

### Paclitaxel Promotes NLRP3 Inflammasome Activation in Murine Macrophages

**Figure 1:**
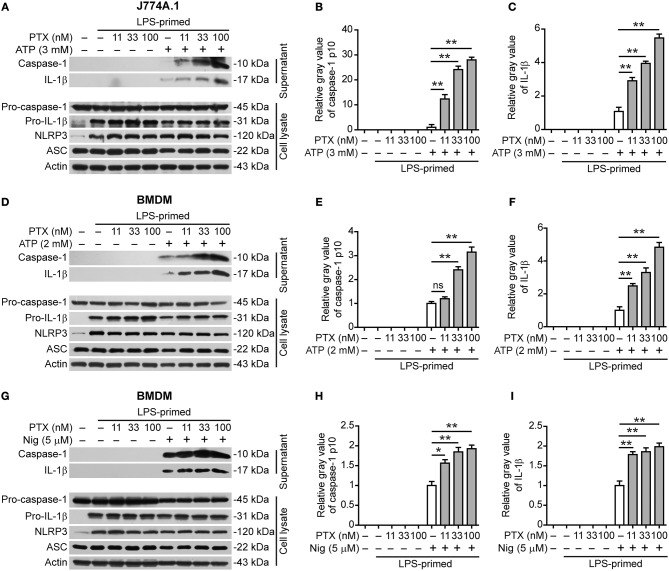
Paclitaxel promoted NLRP3 inflammasome activation in murine macrophages. LPS-primed J774A.1 macrophages were pre-treated paclitaxel for 1 h followed by stimulation with ATP (3 mM) for 1 h, while LPS-primed bone marrow-derived macrophages (BMDMs) were pre-treated with paclitaxel for 1 h followed by stimulation with ATP (2 mM) for 30 min or nigericin (5 μM) for 1 h. **(A,D,G)** Western blot analysis of indicated proteins in the culture supernatants and cell lysates. Actin was used as a loading control. **(B,C,E,F,H,I)** Histograms show the gray value of capase-1p10 (10 kDa) or mature IL-1β (17 kDa) bands in supernatants relative to that of ATP or nigericin group that was set to 1.0, respectively. **(B,C)** Histograms show quantified data from the results presented in **(A)**. **(E,F)** Histograms show quantified data from the results in **(D)**. **(H,I)** Histograms show quantified data from the results in **(G)**. Data were analyzed using the non-parametric Mann–Whitney *U*-test, which are shown as mean ± SD (*n* = 3). ^*^P < 0.05; ^**^P < 0.01; PTX, paclitaxel; Nig, nigericin.

As colchicine acting as a microtubule-destabilizing agent can suppress NLRP3 inflammasome activation (17, 20), we asked whether paclitaxel, a microtubule-stabilizing agent, could influence NLRP3 inflammasome activation. To explore this problem, we assessed the effects of paclitaxel on NLRP3 inflammasome activation in LPS-primed murine J774A.1 or bone marrow-derived macrophages (BMDMs) stimulated with extracellular ATP or nigericin, two canonical NLRP3 inflammasome activators (30). Western blot analysis showed that LPS priming upregulated the expression of NLRP3 and pro-IL-1β proteins (Figure 1). Upon ATP or nigericin stimulation, cleaved caspase-1p10 (10 kDa) and mature IL-1β (17 kDa) were detectable in the culture supernatants of macrophages, indicative of the activation of NLRP3 inflammasome. Interestingly, paclitaxel dose-dependently increased the release of cleaved caspase-1p10 and mature IL-1β into the culture supernatants of J774A.1 cells stimulated with ATP (Figures 1A–C) and of BMDMs stimulated with ATP (Figures 1D–F) or nigericin (Figures 1G–I), indicating that this chemical enhanced NLRP3 inflammasome activation in murine macrophages. Paclitaxel also promoted the activation of non-canonical NLRP3 inflammasome induced by transfecting LPS into macrophages primed with TLR1/2 agonist Pam3CSK4 (Figures S1, S2). However, NLRC4 (activated by flagellin transfection) or AIM2 (activated by poly(dA:dT) transfection) inflammasome activation in Pam3CSK4-primed macrophages were unaffected by paclitaxel pretreatment (Figures S1, S2). Together, these results showed that paclitaxel specifically potentiated both canonical and non-canonical NLRP3 inflammasome activation in murine macrophages.

### Paclitaxel Increases ASC Speck Formation in BMDMs Upon NLRP3 Inflammasome Activation

**Figure 2:**
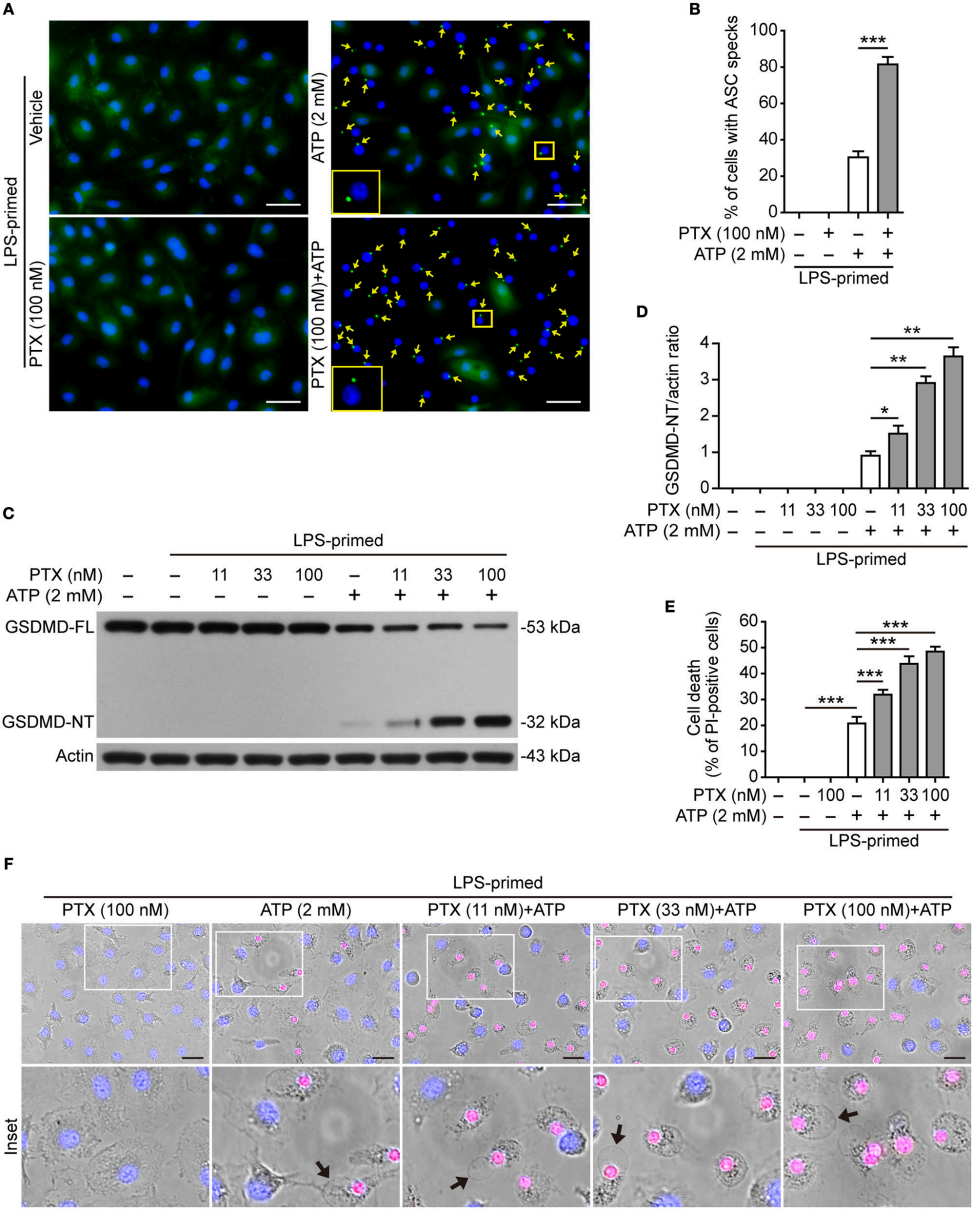
Paclitaxel increased ATP-induced ASC speck formation and pyroptosis in bone marrow-derived macrophages (BMDMs). LPS**-**primed BMDMs were pre-treated with indicated concentrations of paclitaxel for 1 h, followed by incubation with ATP (2 mM) for 30 min without LPS. **(A)** ASC expression and subcellular distribution were revealed by fluorescence microscopy. Representative images (40×) showing ASC (green) subcellular distribution. Nuclei were revealed by Hoechst 33342 (blue). The images for ASC and nuclei were captured under a fluorescence microscope, respectively, and merged together. Yellow arrows indicate ASC specks and the enlarged inset showing a cell containing an ASC speck. Scale bars, 20 μm. **(B)** Percentages of cells with an ASC speck relative to the total cells from 5 random fields each containing ~50 cells. Data were analyzed using the unpaired Student's *t*-test, which are shown as mean ± SD (*n* = 5, one field per well). ^***^P < 0.001. **(C)** Western blotting was used to detect indicated proteins in cell lysates. Actin was used as the loading control. **(D)** Histograms show the quantification of GSDMD-NT levels relative to that of actin. Data were analyzed using the non-parametric Mann–Whitney *U*-test, which are shown as mean ± SD (*n* = 3). **(E, F)** Cell death was assayed by propidium iodide (PI) (red; staining dead cells) and Hoechst 33342 staining (blue; staining all cells) for 10 min. PI-positive cells in 5 randomly chosen fields (one field per well) each containing ~100 cells were quantified (see Figure S3A). **(E)** The percentage of cell death is defined as the ratio of PI-positive relative to all cells (revealed by Hoechst 33342). Data were analyzed using the one-way ANOVA followed by Turkey *post hoc* test, which are shown as mean ± SD (*n* = 5). **(F)** Merged images showing PI (red) and Hoechst 33342 (blue) fluorescence combined with bright-field images. The enlarged insets show the cell morphology. One set of representative images of three independent experiments is shown. Black arrows indicate one dying cell with ballooning from the plasma membrane in each image. Scale bars, 20 μm. GSDMD-FL, full-length GSDMD; GSDMD-NT, GSDMD N-terminal fragment; PTX, paclitaxel. ^*^P < 0.05; ^**^P < 0.01; ^***^P < 0.001.

The assembly and activation of NLRP3 inflammasome requires a key adapter protein named apoptosis-associated speck-like protein containing a caspase recruitment domain (ASC). During the assembly of NLRP3 inflammasome, ASC forms one large speck in each cell by self-oligomerization, which becomes another marker of NLRP3 inflammasome activation (13). To confirm the aforementioned results obtained from Western blot analysis (Figure 1), we next explored whether paclitaxel increased ASC speck formation induced by ATP. Immunofluorescence microscopy analysis showed that ASC distributed evenly in LPS-primed BMDMs, but upon ATP stimulation, ASC specks were observed in ~35% of the cells (Figures 2A,B). Paclitaxel pretreatment greatly increased the numbers of ASC speck induced by ATP stimulation, and in ~85% of the cells each contained one ASC speck near the nucleus (Figure 2A), corroborating that paclitaxel promoted NLRP3 activation in macrophages. These results also suggested that paclitaxel enhanced NLRP3 activation by promoting the assembly of NLRP3 with ASC.

### Paclitaxel Enhances ATP- or Nigericin-Induced Pyroptosis in Macrophages

Active caspase-1 cleaves GSDMD to produce a GSDMD-NT fragment, which binds to and forms pores in the plasma membrane, leading to loss of membrane integrity and a rapid programmed cell death named pyroptosis (15, 23). The pyroptotic cell death is a form of necrosis that can be revealed by propidium iodide (PI) staining. Therefore, we next explored whether paclitaxel could enhance ATP- or nigericin-induced pyroptosis. Consistent with enhanced NLRP3 activation (Figures 1, 2A,B), paclitaxel dose-dependently increased ATP-induced generation of GSDMD-NT (32 kDa) (Figures 2C,D). Concurrent with the production of GSDMD-NT, ~20% of BMDMs were undergoing lytic cell death upon ATP stimulation, and paclitaxel dose-dependently increased the cell death (Figures 2E,F and Figure S3A). The dying cells displayed cell swelling and membrane ballooning (Figure 2F), mirroring the morphological characteristics of pyroptosis. Similarly, paclitaxel also dose-dependently increased lytic cell death and GSDMD cleavage upon nigericin stimulation in LPS-primed BMDMs (Figures S3B–D) or upon ATP stimulation in LPS-primed J774A.1 cells (Figures S4A–C). All these results indicated that paclitaxel promoted NLRP3 inflammasome activation and pyroptosis induced by ATP or nigericin treatment, suggesting its potential in enhancing the innate immune response.

### Paclitaxel Dose- and Time-Dependently Induces α-Tubulin Acetylation

**Figure 3:**
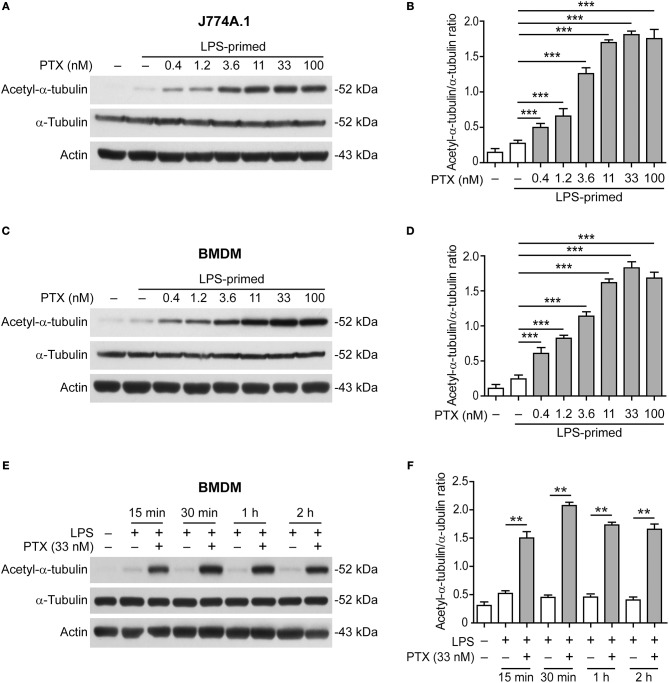
Paclitaxel pretreatment increased the levels of acetylated α-tubulin. LPS-primed J774A.1 macrophages **(A,B)** and BMDMs **(C–F)** were pre-treated with graded concentrations of paclitaxel for 1 h **(A–D)** or with paclitaxel (33 nM) for indicated time periods **(E,F)**. Western blotting was used to assess the levels of indicated proteins in cell lysates **(A,C,E)**. Actin was used as the loading control. Histograms showing the quantification of acetylated α-tubulin relative to total α-tubulin are shown in **(B,D,F)**, respectively. Data were analyzed using the unpaired Student's *t*-test **(B,D)** or non-parametric Mann–Whitney *U*-test **(F)**, which are shown as mean ± SD (*n* = 3). ^**^P < 0.01; ^***^P < 0.001. PTX, paclitaxel.

We next sought to explore how paclitaxel enhanced NLRP3 inflammasome activation. Acetylation of α-tubulin in microtubules has been shown to be critical for ASC trafficking and the assembly of NLRP3 inflammasome (17) while paclitaxel has been shown to induce α-tubulin acetylation (24). We thus assayed whether paclitaxel affected α-tubulin acetylation in LPS-primed macrophages. Western blotting showed that paclitaxel markedly elevated the levels of acetylated α-tubulin in J774A.1 macrophage (Figures 3A,B) and BMDMs in dose- or time-dependent manner (Figures 3C–F), but did not change the levels of total α-tubulin. In addition, paclitaxel-induced acetylation of α-tubulin occurred within 15 min and quickly reached the plateau at 30 min (Figures 3E,F). Although the levels of α-tubulin acetylation in paclitaxel plus ATP or nigericin groups were not further increased as compared to paclitaxel alone, they were much higher than that of ATP or nigericin alone (Figure S5). Enhanced α-tubulin acetylation and NLRP3 inflammasome activation (as indicated by IL-1β release) by paclitaxel were corroborated in human THP-1 macrophages (Figure S6). Together, these results indicated that paclitaxel rapidly and markedly induced α-tubulin acetylation in macrophages, suggesting its involvement in enhanced NLRP3 inflammasome activation.

### Blockade of α-Tubulin Acetylation Attenuates Paclitaxel-Mediated Augmentation of NLRP3 Inflammasome Activation

**Figure 4:**
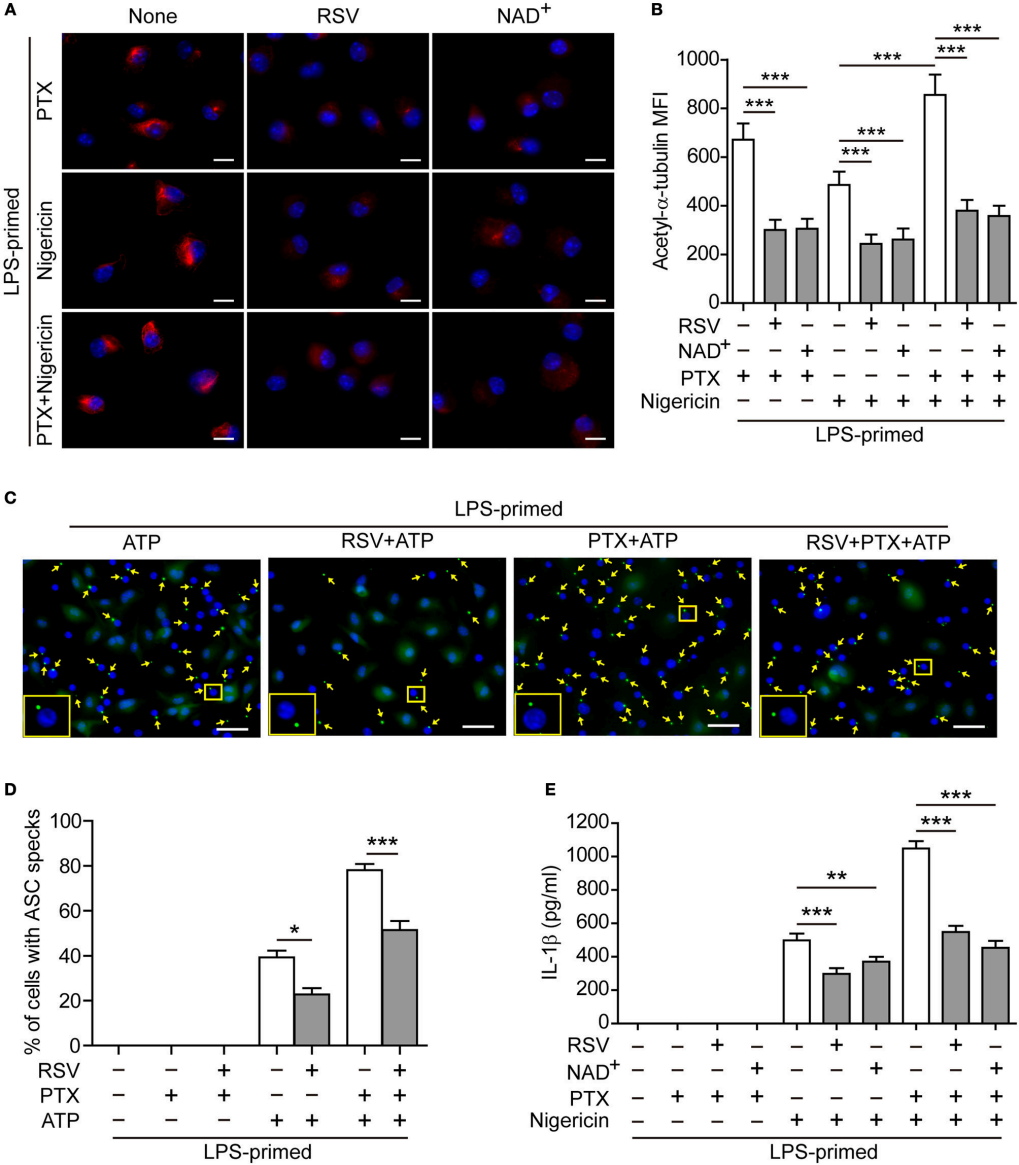
Resveratrol and NAD^+^ suppressed paclitaxel-mediated augmentation of NLRP3 inflammasome activation. **(A,B)** LPS-primed BMDMs were pre-treated without (None) or with resveratrol (5 μM) or NAD^+^ (10 μM) for 30 min before incubation with paclitaxel (33 nM) for 1 h and then stimulation with nigericin (5 μM) for 1 h. After staining with indicated antibodies, the cells were observed by fluorescence microscopy and the images were captured, respectively, and merged together. Representative immunofluorescence images showing acetylated α-tubulin (red) subcellular distribution **(A)**. Nuclei (blue) were revealed by Hoechst 33342. Scale bars, 10 μm. **(B)** Mean of fluorescence intensity (MFI) of acetylated α-tubulin was analyzed by ZEN software. Data were analyzed using the non-parametric Friedman test, which are shown as mean ± SD (*n* = 5)**. (C–E)** LPS-primed BMDMs were pre-treated with resveratrol (5 μM) **(C, D)** or NAD^+^ (10 μM) **(E)** for 30 min prior to paclitaxel (100 nM) treatment for 1 h, followed by incubation with ATP (2 mM) for 30 min. The expression and subcellular distribution of ASC were revealed by the immunofluorescent microscopy. **(C)** Representative images showing ASC (green) subcellular distribution. Nuclei (blue) were revealed by Hoechst 33342. Yellow arrows indicate ASC specks and the enlarged inset showing cells with an ASC speck. Scale bars, 20 μm. **(D)** Percentages of cells with an ASC speck relative to total cells from 5 random fields each containing ~200 cells (see Figure S7). Data were analyzed using the non-parametric Mann–Whitney *U*-test, which are shown as mean ± SD (*n* = 5). **(E)** Levels of soluble IL-1β in culture supernatants were analyzed by cytometric bead array (CBA) assay. Data were analyzed using the one-way ANOVA followed by Turkey *post-hoc* test, which are shown as mean ± SD (*n* = 3). ^*^P < 0.05; ^**^P < 0.01; ^***^P < 0.001; PTX, paclitaxel; RSV, resveratrol.

**Figure 5:**
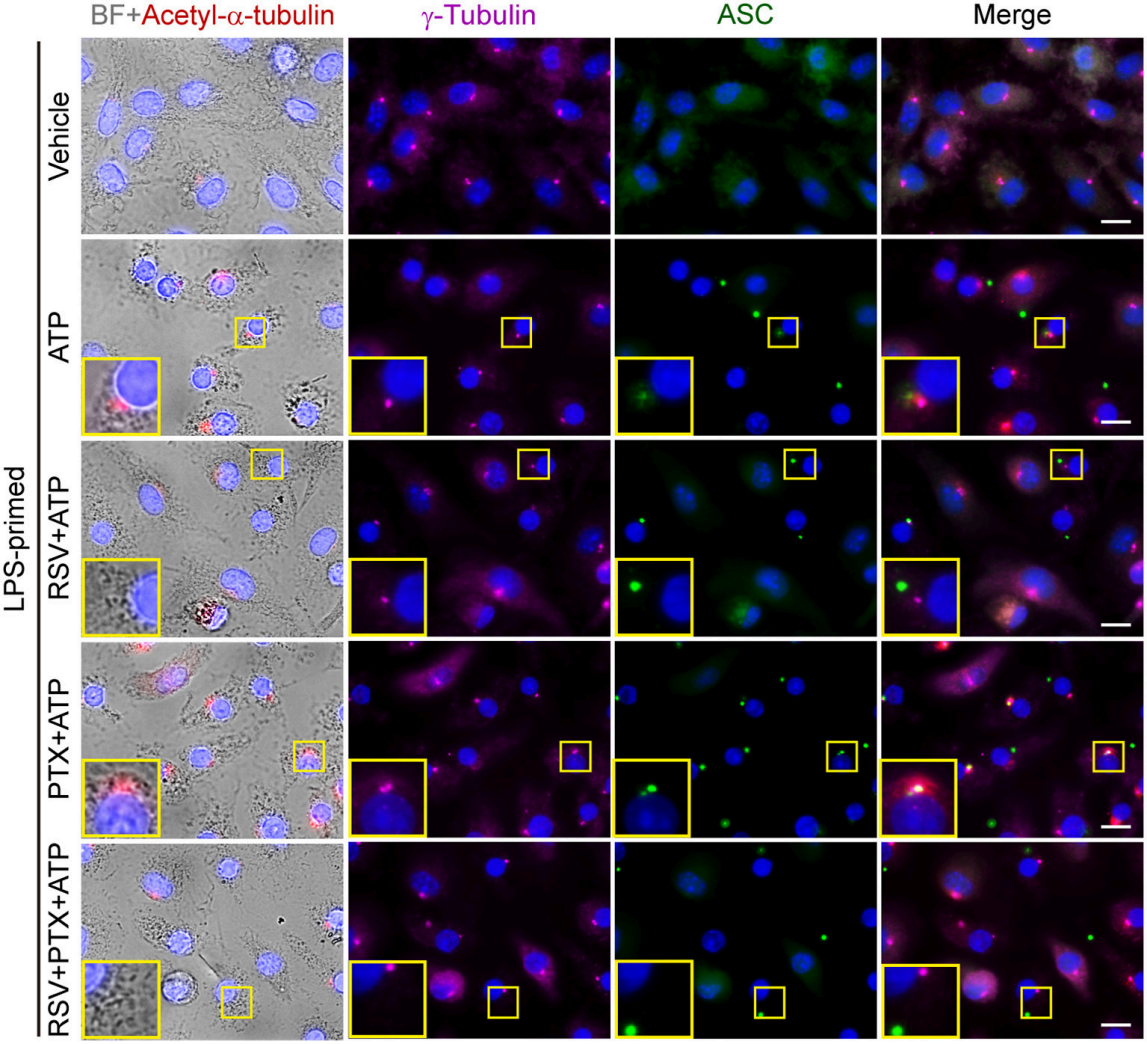
Paclitaxel induced α-tubulin acetylation and promoted the apposition of ASC specks and γ-tubulin upon ATP stimulation. LPS-primed BMDMs were pretreated with resveratrol (100 nM) for 30 min and paclitaxel (100 nM) for 1 h. Then the cells were stimulated with ATP (2 mM) for 30 min. Finally, indicated proteins were stained by indicated antibodies and observed by immunofluorescence microscopy. Bright field (BF) and immunofluoresce images were captured, respectively, while some of which were merged as being indicated. Enlarged insets show the acetylated α-tubulin, γ-tubulin and ASC specks, as well as their subcellular location. Scale bars, 10 μm.

To corroborate the involvement of increased α-tubulin acetylation in mediating paclitaxel-induced augmentation of NLRP3 inflammasome activation, we used pharmacologic agents to block the acetylation of α-tubulin. Immunofluorescence microscopy showed that paclitaxel, nigericin, or their combination induced formation of clusters of acetylated α-tubulin in BMDMs, which were distributed near the perinuclear region of the cell (Figure 4A). Such increased acetylation of α-tubulin was markedly suppressed by resveratrol or NAD^+^ (Figures 4A,B), two activators of NAD^+^-dependent deacetylases Sirt1/Sirt2 (31, 32). Concomitant with the suppression of α-tubulin acetylation, ATP-induced ASC speck formation was significantly decreased by resveratrol, either in the presence or absence of paclitaxel (Figures 4C,D, and Figure S7). Similarly, paclitaxel-induced increase of soluble IL-1β release in the culture supernatants was also suppressed by resveratrol or NAD^+^ (Figure 4E), indicating that these two agents attenuated NLRP3 inflammasome activation in macrophages. Interestingly, accompanying the increase of α-tubulin acetylation, paclitaxel pretreatment enhanced the co-localization of ASC specks with the acetylated α-tubulin and centrioles (revealed by γ-tubulin), whereas resveratrol was able to reverse this process, with fewer ASC specks being co-located with the acetylated α-tubulin and centrioles (Figure 5), suggesting that the apposition of ASC speck and γ-tubulin was mediated by acetylated α-tubulin, and that paclitaxel promoted such a process. Together, these results verified the involvement of paclitaxel-induced α-tubulin acetylation in enhancing NLRP3 inflammasome activation probably by facilitating the assembly of ASC speck.

### The Acetyltransferase MEC-17 Participates in Paclitaxel-Mediated Acetylation of α-Tubulin and Augmentation of NLRP3 Inflammasome Activation

**Figure 6:**
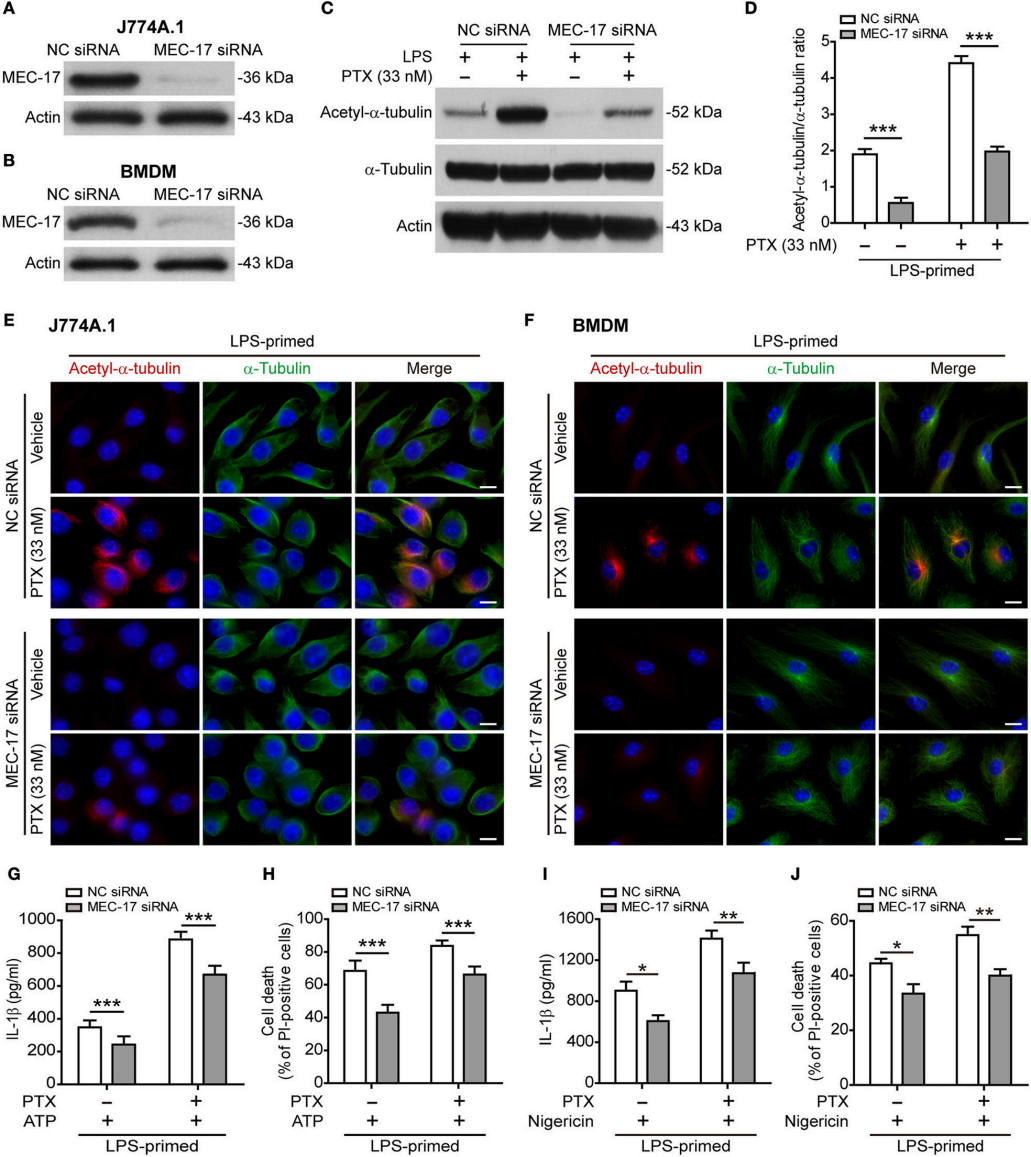
*MEC-17* knockdown attenuated paclitaxel-mediated augmentation of NLRP3 inflammasome activation and pyroptosis. J774A.1 macrophages and BMDMs were transfected with negative control (NC) siRNA or *MEC-17* siRNA for 72 h and were used for the following experiments. **(A,B)** The levels of MEC-17 expression in J774A.1 **(A)** or BMDMs **(B)** were detected by Western blotting. **(C–F)** LPS-primed J774A.1 macrophages were treated with paclitaxel (33 nM) for 1 h. The expression of acetylated α-tubulin and α-tubulin were revealed by Western blotting **(C)** and the relative gray values of acetylated α-tubulin to α-tubulin were quantified **(D)**. Data are shown as mean ± SD (*n* = 3). Immunofluorescence microscopy revealed the expression of acetylated α-tubulin (red) and α-tubulin (green) in J774A.1 cells **(E)** or in BMDMs **(F)**. Nuclei were stained with Hoechst 33342 (blue). Scale bars, 10 μm. **(G–J)** Cells were primed with LPS (500 ng/ml for 4 h) before treated with paclitaxel (33 nM) for 1 h, followed by incubation with ATP (3 mM for 1 h) in J774A.1 **(G,H)** or nigericin (5 μM for 1 h) in BMDMs **(I,J)**. The levels of soluble IL-1β were detected by cytometric bead array **(G,I)**. Cell death was measured by staining with propidium iodide (PI) and Hoechst 33342 together for 10 min. PI-positive cells were quantified by counting 5 randomly chosen fields (one field per well) containing around 100 cells each **(H,J)**. Data were analyzed using the unpaired Student's *t*-test, which are shown as mean ± SD (*n* = 5). ^*^P < 0.05; ^**^P < 0.01; ^***^P < 0.001. PTX, paclitaxel.

Published studies have shown that MEC-17 (also known as α-tubulin acetyltransferase 1, αTAT1) is responsible for the acetylation of α-tubulin (33, 34), thus being involved in NLRP3 inflammasome activation (17). To test whether MEC-17 was involved in paclitaxel-mediated augmentation of NLRP3 activation, we knocked down *MEC-17* expression by using siRNA. Western blot analysis showed that the expression of MEC-17 was decreased by ~90% after siRNA knockdown of *MEC-17* as compared to negative control (Figures 6A,B). As expected, *MEC-17* knockdown markedly suppressed the levels of both basal (without paclitaxel treatment) and paclitaxel-induced α-tubulin acetylation in LPS-primed J774A.1 cells (Figures 6C,D). Immunofluorescence microscopy also revealed that paclitaxel-induced α-tubulin acetylation was markedly attenuated by *MEC-17* siRNA as compared to NC siRNA treatment in LPS-primed J774A.1 and BMDM cells, whereas α-tubulin distribution was largely unaffected (Figures 6E,F). Furthermore, NLRP3 activation (indicated by IL-1β release) and lytic cell death (indicated by PI staining) was induced by ATP in J774A.1 cells, but was significantly suppressed in those cells with *MEC-17* knockdown, either in the presence or absence of paclitaxel (Figures 6G,H). Similarly, *MEC-17* knockdown significantly suppressed nigericin-induced soluble IL-1β release and cell death in LPS-primed BMDMs, either in the presence or absence of paclitaxel (Figures 6I,J). Together, these results indicated that MEC-17 was involved in paclitaxel-induced augmentation of NLRP3 inflammasome activation by increasing acetylation of α-tubulin.

### Epothilone B, Like Paclitaxel, Does Not Influence Macrophage Priming but Does Enhance NLRP3 Inflammasome Activation

**Figure 7:**
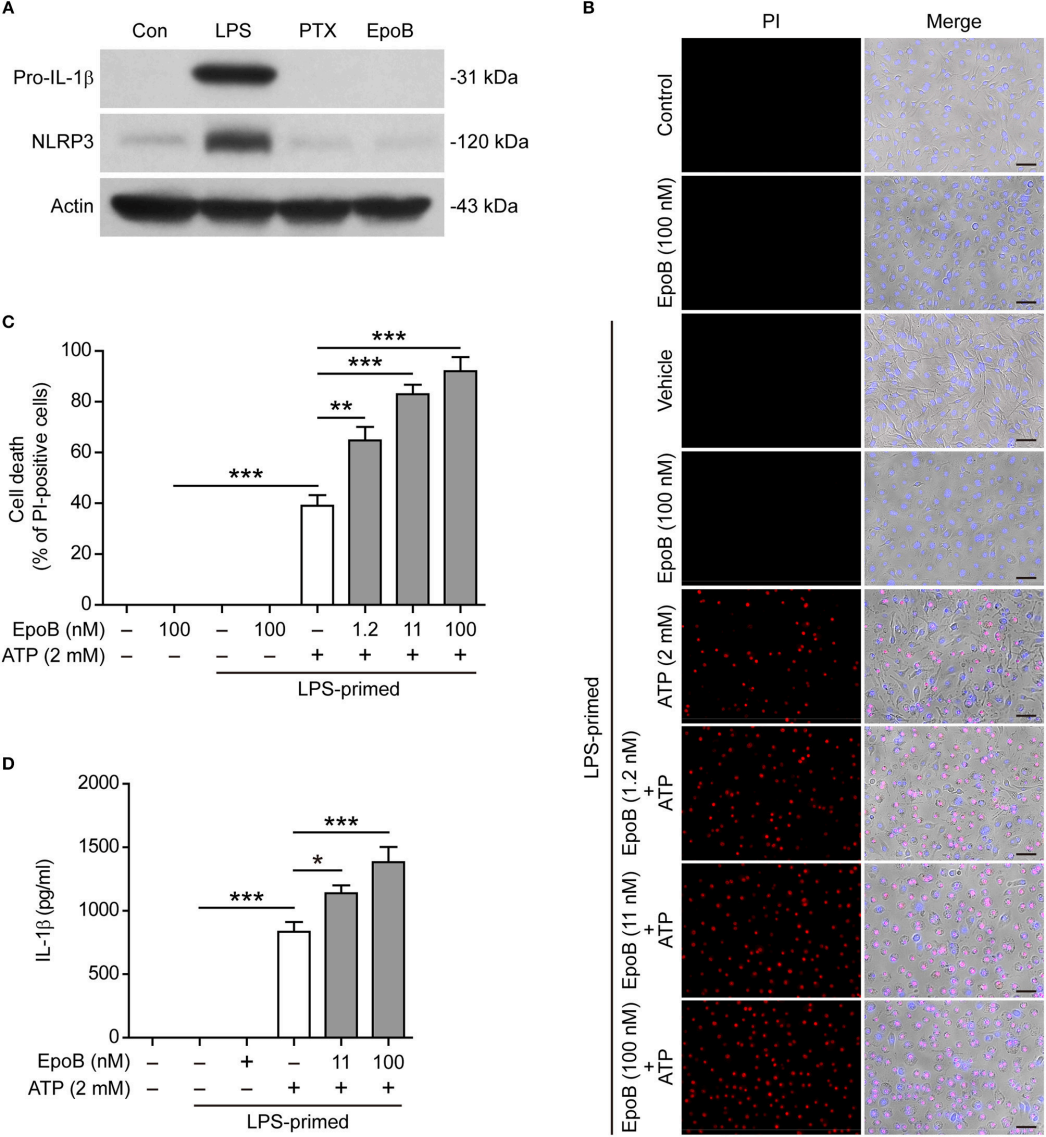
Paclitaxel did not influence macrophage priming in the process of NLRP3 inflammasome activation. **(A)** BMDMs were treated with LPS (500 ng/ml), paclitaxel (100 nM) and epothilone B (100 nM) for 4 h. Indicated proteins in the cell lysates were analyzed by Western blotting. Actin was used as a loading control. **(B–D)** LPS**-**primed BMDMs were pre-treated with graded doses of epothilone B for 1 h, followed by incubation with ATP (2 mM) for 30 min. **(B,C)** Cells were stained by Hoechst 33342 (blue; for all cells) and propidium iodide (PI) (red; for dead cells) for 10 min. **(B)** All images were captured by fluorescence microscopy, and the merged images show PI and Hoechst 33342 fluorescence with bright-field images. One set of representative images of three independent experiments are shown. Scale bars, 50 μm. **(C)** PI-positive cells in 5 randomly chosen fields (one field per well) each containing ~100 cells were quantified. The percentage of cell death is defined as the ratio of PI-positive relative to all (revealed by Hoechst 33342) cells. **(D)** The levels of soluble IL-1β in culture supernatants were analyzed by cytometric bead array (CBA) assay. **(C,D)** Data were analyzed using the one-way ANOVA followed by Turkey *post-hoc* test, which are shown as mean ± SD (*n* = 5). ^*^P < 0.05; ^**^P < 0.01; ^***^P < 0.001; PTX, paclitaxel; EpoB, Epothilone B.

Next, we explored whether epothilone B, another microtubule-stabilizing drug that is functionally similar to paclitaxel but has a distinct molecular structure (35), also enhanced NLRP3 inflammasome activation. The results showed that, similar to paclitaxel, single epothilone B did not induce the expression of pro-IL-1β and NLRP3 in unprimed BMDMs (Figure 7A), but it evidently enhanced pyroptosis (Figures 7B,C) and IL-1β release (indicative of NLRP3 inflammasome activation) (Figure 7D) in LPS-primed BMDMs. Interestingly, epothilone B also dose-dependently induced α-tubulin acetylation in LPS-primed BMDMs (Figure S8), suggesting that epothilone B and paclitaxel shared a common mechanism in enhancing NLRP3 inflammasome activation.

Previous studies showed that paclitaxel was an LPS mimetic that can bind with Toll-like receptor 4 (TLR4) to induce MyD88/NF-κB signaling (36, 37). However, it is unclear whether this activity of paclitaxel accounts for its action in enhancing NLRP3 inflammasome activation and pyroptosis. To clarify this issue, we first explored whether paclitaxel had a similar effect like LPS in priming macrophages. Western blot analysis showed that LPS induced the expression of pro-IL-1β and NLRP3 that are requisite for NLRP3 inflammasome assembly but paclitaxel did not (Figure 7), indicating that paclitaxel had no macrophage-priming activity in the process of inflammasome activation.

### Paclitaxel Administration Enhances the Innate Immune Response Against Bacterial Infection in Mice

**Figure 8:**
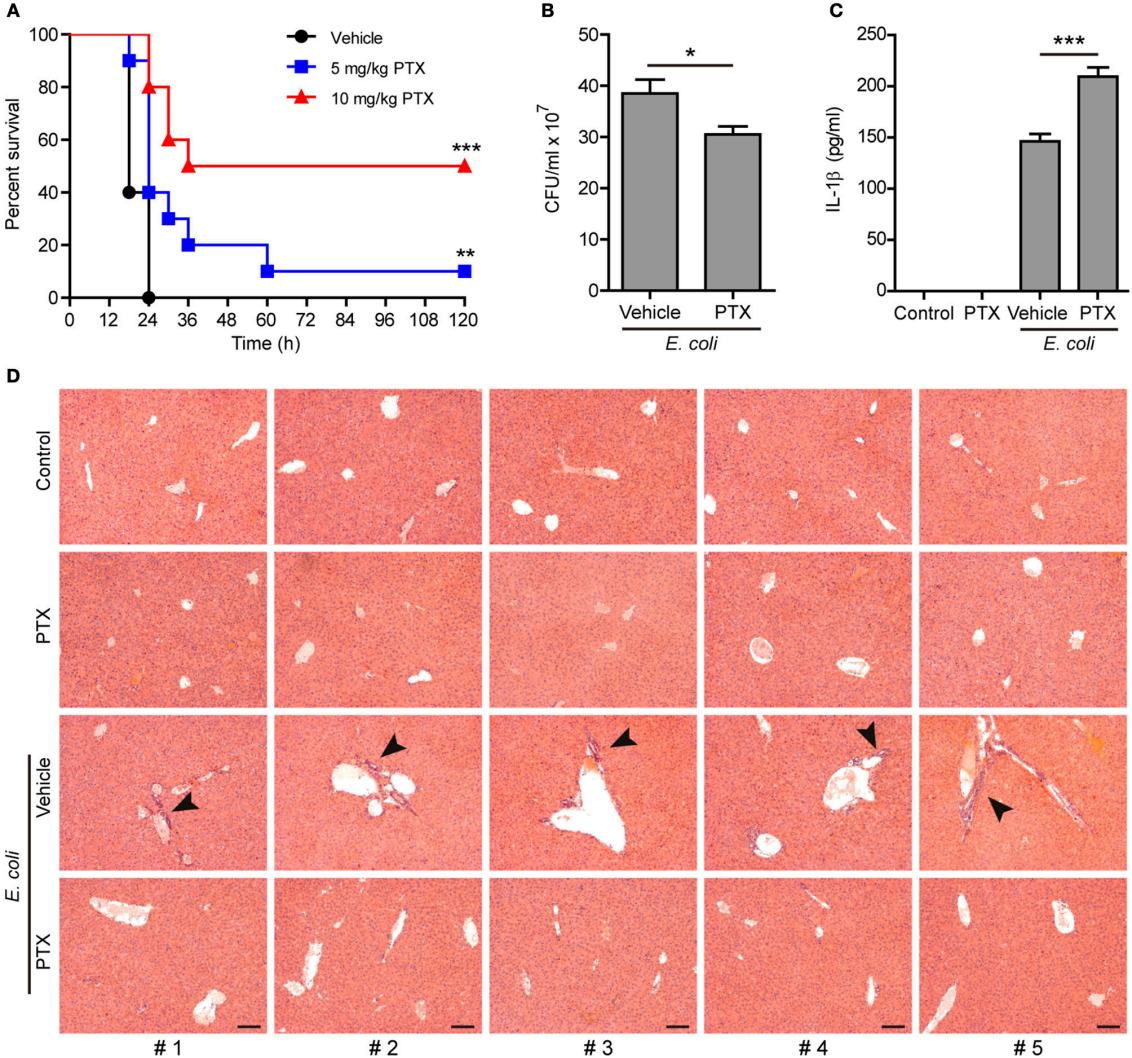
Paclitaxel administration prolonged mouse survival during bacterial infection. **(A)** Mice were injected (i.p.) with paclitaxel (5 and 10 mg/kg body weight) or vehicle (PBS) 1 h before peritoneal injection with viable *Escherichia coli* (2 × 10^9^ CFU/mouse). Mouse survival was monitored every 6 h for 5 consecutive days. Kaplan–Meier survival curves were used to analyze the data (10 mice per group). The significance was evaluated by the log-rank (Mantel–Cox) test. Three independent experiments were performed and one representative set of data were shown. ^**^P < 0.01; ^***^P < 0.001. **(B–D)** Mice were injection (i.p.) with paclitaxel (10 mg/kg body weight) or vehicle (PBS) 1 h before peritoneal injection with viable *E. coli* (1 × 10^9^ CFU/mouse) for 8 h. Bacterial counts in the peritoneal cavity was measured by using an ultraviolet-visible spectrophotometer, and the corresponding CFUs were determined on LB media agar plates **(B)**. The serum levels of IL-1β were measured by cytometric bead array (five mice per group) **(C)**. **(B,C)**, Data were analyzed using the unpaired Student's *t*-test, which are shown as mean ± SD (*n* = 5). ^*^P < 0.05; ^***^P < 0.001. Representative images of hematoxylin and eosin (H&E) staining of the liver section are shown and arrowheads indicated infiltrated inflammatory cells **(D)**. The numbers at the bottom indicate mouse number. Scale bars, 100 μm. PTX, paclitaxel.

As NLRP3 inflammasome activation represents critical innate defense mechanism against bacterial infection (38), we next explored the functional relevance of paclitaxel-mediated augmentation of NLRP3 activation in a mouse model of bacterial infection. To this end, mice were intraperitoneally injected with paclitaxel (5 and 10 mg/kg body weight) or vehicle (PBS) 1 h before peritoneal injection with a lethal dose of viable *E. coli* (2 × 10^9^ CFU/mouse). All vehicle-treated mice were succumbed to such a lethal dose of *E. coli* infection within 24 h, whereas ~10% (in 5 mg/kg paclitaxel group) and 50% (in 10 mg/kg paclitaxel group) of paclitaxel-treated mice survived the experimental period of observation (120 h), respectively (Figure 8A). In a parallel experiment, the peritoneal bacterial burden and IL-1β levels in the serum were evaluated at 8 h post infection. The bacterial burden in the peritoneal fluids was significantly reduced in paclitaxel group as compared to vehicle group (Figure 8B). Moreover, paclitaxel administration significantly increased the serum levels of IL-1β in the mouse model of bacterial infection (Figure 8C). Consistent with reduced bacterial burden and increased mouse survival, paclitaxel-treated mice displayed decreased infiltration of inflammatory cells in the liver as compared with vehicle group (Figure 8D). These results indicated that paclitaxel potentiated the innate immune response against bacterial infection probably by increasing NLRP3 inflammasome activation *in vivo* in mice.

## Discussion

It has been demonstrated that paclitaxel can ameliorate LPS-induced kidney injury and improve animal survival in a mouse model of LPS-induced sepsis (39, 40). Paclitaxel can also attenuate sepsis-induced liver injury (41). Although some studies have implicated paclitaxel in regulating NLRP3 inflammasome activation (25), the underlying mechanism has not been completely elucidated. In this study, we demonstrated that paclitaxel enhanced NLRP3 inflammasome activation and pyroptosis, leading to increased IL-1β release and enhanced anti-bacterial response *in vivo*. Consistent with this, paclitaxel treatment significantly improved animal survival in a mouse model of bacterial infection. Such immunomodulatory activity of paclitaxel suggests its potential application, beyond in cancer therapy, in infectious diseases.

Intriguingly, the assembly of NLRP3 inflammasome relies on the cargo trafficking machinery of microtubule tracks (17, 18). Microtubules are composed of α- and β-tubulin subunits, serving as a major cytoskeleton to maintain cell morphology. They are also tracks for cargo trafficking (42). Several manners of microtubule post-translational modifications have been reported (43). For example, the α-subunit can be acetylated by α-tubulin acetyltransferase 1 (αTAT1/MEC-17) at lysine 40, which is located inside the microtubule (33, 43), while SIRT2 serves as an α-tubulin deacetylase (44). Although acetylation of α-tubulin does not change the assembly and morphology of microtubules, it increases the mechanical stability and flexibility of the microtubules, accelerating their shrinkage and thus promoting vesicle transport (45). Upon α-tubulin acetylation, microtubules tend to form bundles, which enhance the motor functions of dynein and kinesin in transporting cargoes (46, 47). During the assembly of NLRP3 inflammasome, the adaptor protein ASC is located on mitochondria that are transported as cargoes along the microtubule to the minus end, where the centrioles dwell, forming the mitochondrion associated ER membrane (MAM) (17). In line with this, the apposition of NLRP3 specks, γ-tubulin (indicative of centrioles) and the microtubule-affinity regulating kinase 4 (MARK4), a microtubule-associated protein (MAP) regulating microtubule dynamics, has been observed upon NLRP3 inflammasome activation (18). Interestingly, α-tubulin is acetylated upon ATP and nigericin treatment, while the NAD^+^-dependent deacetylase SIRT2 was suppressed by them due to reduced NAD^+^ concentration in this process (17). Two activators of SIRT2, resveratrol and NAD^+^, suppress the approximation of ASC to NLRP3, thus inhibiting the assembly of NLRP3 inflammasome (48). Therefore, α-tubulin acetylation seems necessary for the trafficking of inflammasome components including ASC to the perinuclear region, thus accelerating NLRP3 inflammasome assembly. Consistent with previous studies (24), we demonstrated that paclitaxel is a strong inducer of α-tubulin acetylation as a low concentration of paclitaxel (0.4 nM) was sufficient to induce α-tubulin acetylation in LPS-primed macrophages (see Figure 3). Based on the studies mentioned above together with our observation, we proposed that induction of α-tubulin acetylation by paclitaxel facilitated the assembly of NLRP3 inflammasome. Indeed, paclitaxel treatment led to increased formation of ASC specks upon ATP or nigericin stimulation, and most of the ASC specks were observed apposition with γ-tubulin, whereas inhibition of the α-tubulin acetylation by inhibitors (NAD^+^ or resveratrol) greatly reduced the ASC speck formation induced by paclitaxel plus ATP (see Figures 4A, 5). This was likely due to that resveratrol and NAD^+^ prevented the minus end-oriented trafficking of ASC along the microtubule track by decreasing α-tubulin acetylation, thus preventing the apposition of ASC specks with γ-tubulin. In line with the reduction of ASC specks, paclitaxel-augmented NLRP3 inflammasome activation was also inhibited by resveratrol, NAD^+^, or *MEC-17* siRNA knockdown. Our data suggest that acetylated α-tubulin-mediated apposition of ASC and γ-tubulin is required for NLRP3 inflammasome activation, and that paclitaxel, via inducing α-tubulin acetylation, promotes ASC trafficking and speck formation during the assembly of NLRP3 inflammasome.

Although paclitaxel strongly induces α-tubulin acetylation as revealed by previous studies (24) and ours, the underlying mechanism is not fully elucidated. Previous reports have indicated that paclitaxel binds to β-tubulin and stabilize the microtubule (49). As the lysine 40 residue is buried inside the lumen of microtubule, paclitaxel binding to the microtubule may make the αTAT1 easier to access the lumen (lysine 40), thus leading to increased acetylation of α-tubulin. Alternatively, paclitaxel may block the deacetylation process by SIRT2 via yet-unknown mechanism. Interestingly, previous reports have indicated that berberine also induces α-tubulin acetylation in tumor cells (50). However, berberine did not induce α-tubulin acetylation in the context of our experiment (i.e., in LPS-primed macrophages, data not shown) at the concentrations that enhance NLRP3 inflammasome activation as revealed by us previously (51). Several studies have indicated that paclitaxel induces mitochondrial reactive oxidative species (ROS) (52), which is an inducer of α-tubulin acetylation (53). Interestingly, paclitaxel in combination of lentinan (a polysaccharide isolated from mushroom) triggers ROS production in A549 cells and enhances apoptosis by activating ROS-TXNIP-NLRP3 inflammasome (54). These studies suggest that paclitaxel enhanced the NLRP3 inflammasome activation by induction of ROS, although paclitaxel-induced ROS (if there was) was not detected in the present study. Indeed, reducing ROS production by resveratrol has contributed to its action to suppress NLRP3 inflammasome activation (48). There are also studies indicating that paclitaxel is an LPS mimetic, which binds to MD-2/TLR-4 and results in the activation or suppression of NF-κB activation (40). However, our data showed that paclitaxel *per se* did not induce the expression of NLRP3 and pro-IL-1β (indicators of macrophage priming) as LPS does, nor did it influence the levels of these proteins induced by LPS (see Figure 1). Moreover, paclitaxel did not influence the AIM2 and NLRC4 inflammasome activation induced by poly(dA:dT) and flagellin, respectively. Interestingly, epothilone B, another microtubule-stabilizing molecular like paclitaxel but with a distinct molecular structure (35), showed similar activities as paclitaxel in enhancing pyroptosis and IL-1β release as well as inducing α-tubulin acetylation in LPS-primed macrophages. Therefore, it is unlikely that paclitaxel enhanced NLRP3 inflammasome activation by increased macrophage priming, but by sharing a common mechanism with epothilone B to induce α-tubulin acetylation.

Interestingly, in contrast to the action of paclitaxel, colchicine has been shown to suppress NLRP3 inflammasome activation in macrophages stimulated by nigericin, ATP or monosodium urate crystal (MSU) (17, 20). Mechanistically, colchicine inhibited the activation of NLRP3 inflammasome by decreasing the levels of acetylated α-tubulin induced by the stimulators (17). These studies have explained the pharmacological action of colchicine in clinical treatment of gout (55), an inflammatory disease induced by MSU (20). Indeed, the anti-gout activity of colchicine is likely due to suppression of the inflammasome activation by MSU (21). Considering that colchicine is a microtubule-destabilizing agent (56) while paclitaxel being a microtubule-stabilizing agent (24), the above-mentioned studies together with our present data suggest that targeting microtubules may either suppress or booster NLRP3 inflammasome activation in terms of differential actions on the microtubule cytoskeleton.

Previous studies have indicated that full activation of NLRP3 inflammasome is required for efficient elimination of invaded pathogens, and loss of the NLRP3 inflammasome machinery, including genetic deficiency of *IL-1r, caspase-1* and/or *-11*, aggravates the infectious diseases and increases animal death (57). Mice with both *caspase-1* and *-11* deficiencies, thus lacking mature IL-1β and IL-18, lose the capacity to recruit neutrophils and natural killer (NK) cells to the infectious site, and they can even succumb to common environmental bacteria (58). Mature IL-1β is produced by active caspase-1 upon inflammasome activation and it is a strong chemoattractant for neutrophils, one of the most important immune cells that engulf and kill pathogens (59). By binding to the receptor IL-1R, IL-1β participates in macrophage differentiation and activation (60), increasing their capacity for presenting pathogenic antigens to T lymphocytes (61). It also stimulates T lymphocytes to differentiate into T helper (Th) 17/Th1 cells (62) and is required for B cell activation, proliferation and antibody production (63). Therefore, increased IL-1β secretion by paclitaxel upon inflammatory stimuli, suggestive of enhanced innate and adaptive immunity, is likely beneficial for bacterial clearance. Indeed, our study revealed that paclitaxel promoted the clearance of bacteria and improved animal survival, accompanied by alleviated infiltration of inflammatory cells in the liver. In further support of this notion, we have reported that another Chinese herbal ingredient berberine with similar activities to paclitaxel in enhancing NLRP3 inflammasome activation exhibited significant anti-bacterial activity through enhancing the innate immunity of the host (51). Therefore, augmentation of NLRP3 inflammasome activation by paclitaxel via inducing α-tubulin acetylation makes it potential to be developed as a new antimicrobial and immunomodulatory medicine.

As a chemotherapeutic agent, paclitaxel can induce many side effects, including hair loss, bone marrow suppression, numbness, allergic reactions, muscle pains, diarrhea, heart problems, increased risk of infection, and lung inflammation (64, 65). But the doses used in clinic seem higher than those used in our study. More importantly, paclitaxel alone neither induced NLRP3 inflammasome activation nor caused cell death in LPS-primed macrophages. Besides, our experiment showed that paclitaxel did not induce acute tissue damage in mice without bacterial infection (Figure 8D). Therefore, the side effects of paclitaxel in clinic may not relate to its activity in enhancing NLRP3 activation.

In summary, we demonstrated that paclitaxel was able to potentiate innate immune responses, including NLRP3 inflammasome activation, IL-1β release and pyroptosis, reducing bacterial burden and improving animal survival. The data suggest that paclitaxel may have potential application in combating pathogenic infection and other inflammatory diseases. In particular, this property of paclitaxel suggests its potential application in treatment of sustained infection-related lesions such as sores, furuncle and beriberi. Further investigation is warranted to verify the *in vivo* effects of paclitaxel in appropriate pre-clinical animal models of such diseases.

## Ethics Statement

All animal experiments were performed according to the guidelines for the care and use of animals approved by the Committee on the Ethics of Animal Experiments of Jinan University.

## Author Contributions

QZe, FY, CL, and LX performed *in vitro* studies. CL, LX, FM, CZe, and CZh conducted animal studies. CL and QZe analyzed the data. DO, QZh and XH supervised the study. DO and XH wrote the paper.

### Conflict of Interest Statement

The authors declare that the research was conducted in the absence of any commercial or financial relationships that could be construed as a potential conflict of interest.
