# Supplementary material for: Paclitaxel Enhances the Innate Immunity by Promoting NLRP3 Inflammasome Activation in Macrophages
Source: Front Immunol. 2019 Jan 29;10:72. doi: 10.3389/fimmu.2019.00072 (PMC6361797; doi:10.3389/fimmu.2019.00072)
Supplement: Supplementary file 1 [file Data_Sheet_1.docx]

**Supplementary Materials**


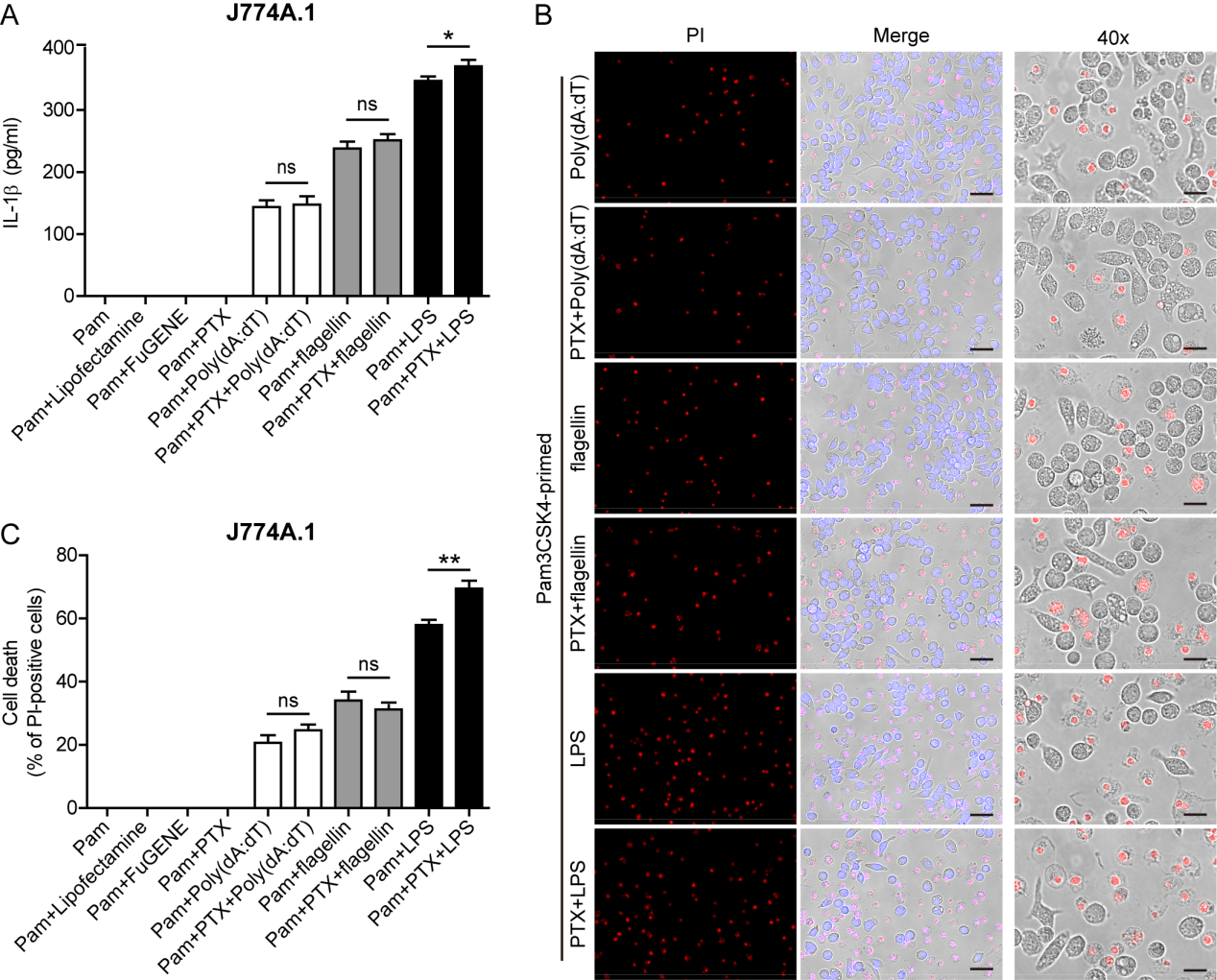


**Figure S1. Paclitaxel had no effects on NLRC4 inflammasome and AIM2 inflammasome in J774A.1 macrophages.** J774A.1 macrophages were primed with Pam3CSK4 (1 μg/ml) for 4 h followed by paclitaxel (100 nM) treatment for 1 h and then transfection with poly (dA:dT) (2 μg/ml), flagellin (0.5 μg/ml) or LPS (2.5 μg/ml) for 16 h. **(A)** The levels of soluble IL-1β in culture supernatants were analyzed by cytometric bead array (CBA) assay. Data were analyzed using the non-parametric Mann–Whitney U test, which are shown as mean ± SD (*n* = 3). **P*< 0.05; ns, not significant; Cells were stained by Hoechst 33342 (blue; for all cells) and propidium iodide (PI) (red; for dead cells) for 10 min. **(B)** All images were captured by fluorescence microscopy, and the merged images show PI and Hoechst 33342 fluorescence with bright-field images. Representative images showing PI fluorescence (red) combined with bright-field images. Scale bars, 50 μm. Right panels show merged images (40×) showing PI fluorescence (red) combined with bright-field images. Scale bars, 20 μm. One set of representative images of three independent experiments are shown. **(C)** PI-positive cells in 5 randomly chosen fields (one field per well) each containing ~100 cells were quantified. The percentage of cell death is defined as the ratio of PI-positive relative to all (revealed by Hoechst 33342) cells. Data were analyzed using the non-parametric Mann–Whitney U test, which are shown as mean ± SD (n =5). ***P* < 0.01; ns, not significant; PTX, paclitaxel; Pam, Pam3CSK4.


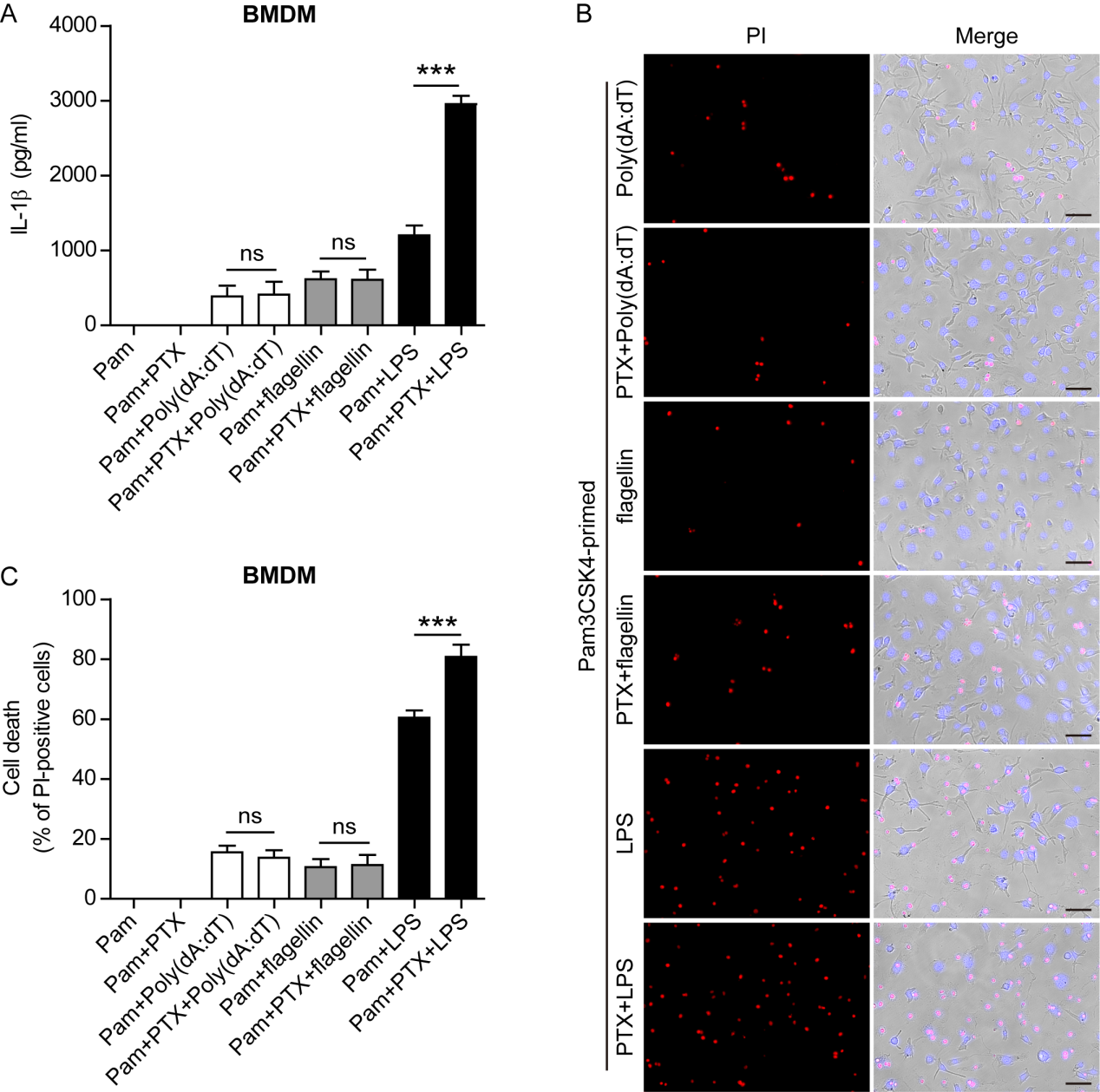


**Figure S2. Paclitaxel had no effects on NLRC4 inflammasome and AIM2 inflammasome in BMDMs.** BMDMs were primed with Pam3CSK4 (1 μg/ml) for 4 h followed by paclitaxel (100 nM) treatment for 1 h and then transfection with poly (dA:dT) (2 μg/ml), flagellin (0.5 μg/ml) or LPS (2.5 μg/ml) for 16 h. (**A**) The levels of soluble IL-1β in culture supernatants were analyzed by cytometric bead array (CBA) assay. Data were analyzed using unpaired Student t-test, which are shown as mean ± SD (n = 3). ****P*< 0.001; ns, not significant; Cells were stained by Hoechst 33342 (blue; for all cells) and propidium iodide (PI) (red; for dead cells) for 10 min. (**B**) All images were captured by fluorescence microscopy, and the merged images show PI and Hoechst 33342 fluorescence with bright-field images. Scale bars, 50 μm. One set of representative images of three independent experiments are shown. (**C**) Histograms showing ratios of PI-positive cells quantified by counting 5 randomly chosen fields (one field per well) containing ~100 cells each. The percentage of lytic cell death is defined as the ratio of PI-positive cells relative to all (revealed by Hoechst 33342) cells. Data were analyzed using unpaired Student’s t-test, which are shown as mean ± SD (n =5). ****P* < 0.001; ns, not significant; PTX, paclitaxel; Pam, Pam3CSK4.


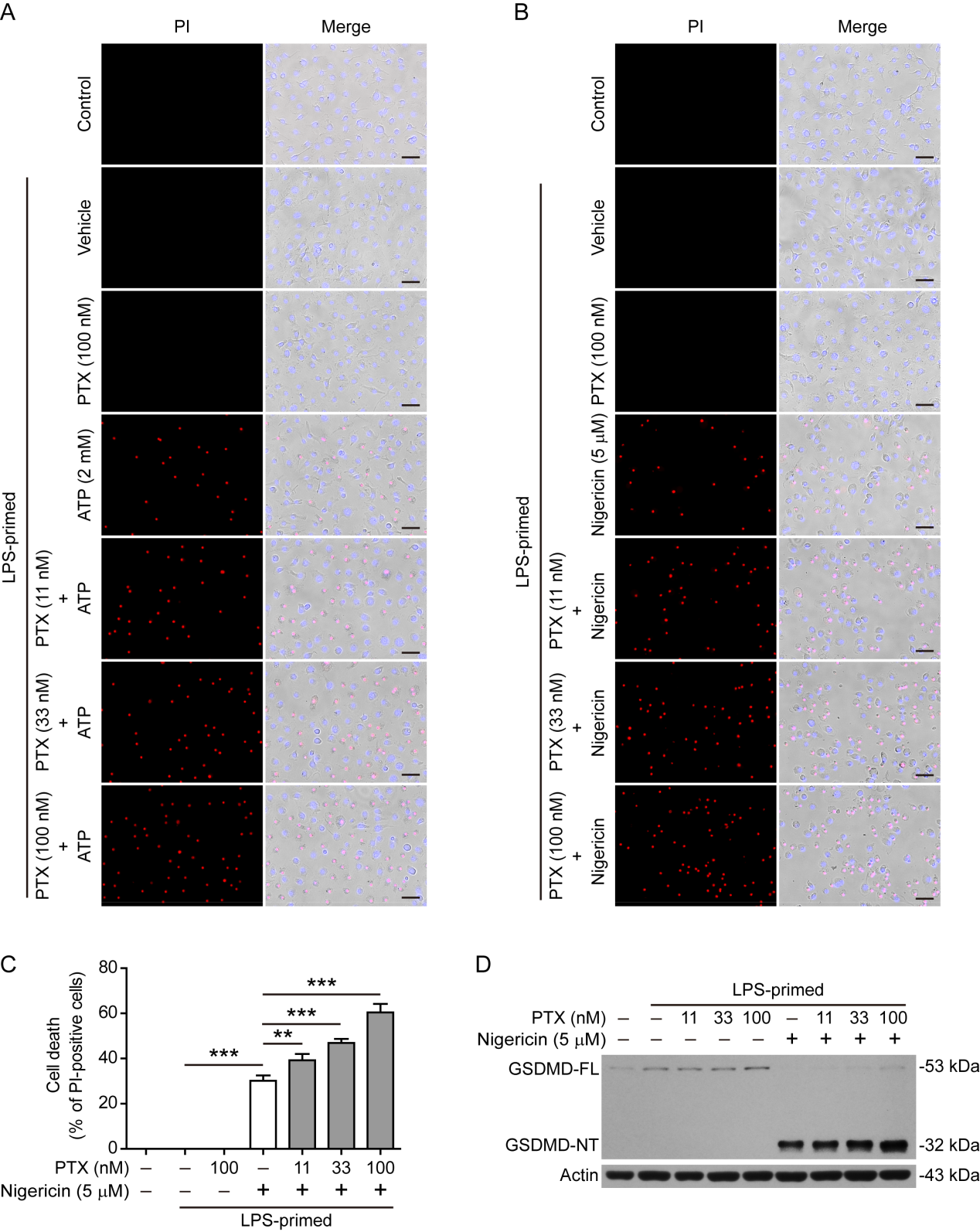


**Figure S3. Paclitaxel increased ATP- or nigericin-induced pyroptosis in BMDMs.** BMDMs were first primed with LPS (500 ng/ml) for 4 h, and then pre-treated with graded doses of paclitaxel for 1h, followed by incubation with ATP (2 mM) for 30 min (**A**) or nigericin (5 μM) for 1 h (**B, C, D**). (**A, B**) Cells were stained by Hoechst 33342 (blue; for all cells) and propidium iodide (PI) (red; for dead cells) for 10 min. All images were captured by fluorescence microscopy, and the merged images show PI and Hoechst 33342 fluorescence with bright-field images. One set of representative images of three independent experiments are shown. Scale bars, 50 μm. (**C**) The percentage of cell death is defined as the ratio of PI-positive relative to all cells (revealed by Hoechst). Data Data were analyzed using the One-way ANOVA followed by Turkey post hoc test, which are shown as mean ± SD (n = 5). ***P* < 0.01; ****P* < 0.001. (**D**) Western blotting was used to detect indicated proteins in cell lysates. Actin was used as the loading control. GSDMD-FL, full-length GSDMD; GSDMD-NT, GSDMD N-terminal fragment; PTX, paclitaxel.


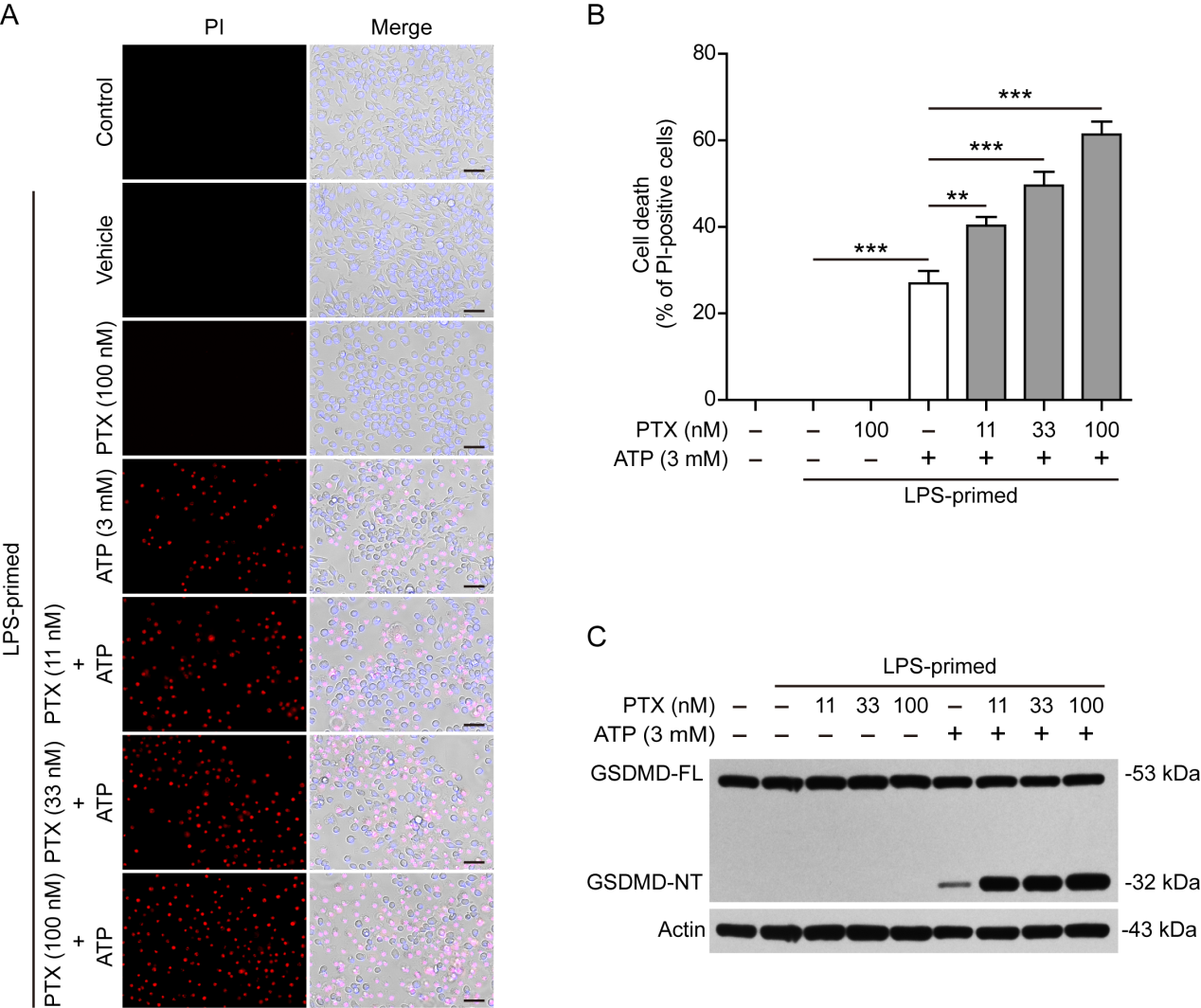


**Figure S4. Paclitaxel enhanced ATP-induced pyroptosis dependently on GSDMD cleavage in J774A.1 macrophages.** LPS-primed J774A.1 macrophages were pre-treated paclitaxel for 1 h followed by stimulation with ATP (3 mM) for 1 h. (**A**) Cells were stained by Hoechst 33342 (blue; for all cells) and propidium iodide (PI) (red; for dead cells) for 10 min. All images were captured by fluorescence microscopy, and the merged images show PI and Hoechst 33342 fluorescence with bright-field images. One set of representative images of three independent experiments are shown. Scale bars, 50 μm. (**B**) The percentage of cell death is defined as the ratio of PI-positive cell to total (revealed by Hoechst) cells. Data were analyzed using the One-way ANOVA followed by Turkey post hoc test, which are shown as mean ± SD (n = 5). ***P* < 0.01; ****P* < 0.001. (**C**) Western blotting was used to detect indicated proteins in cell lysates. Actin was used as the loading control. GSDMD-FL, full-length GSDMD; GSDMD-NT, GSDMD N-terminal fragment; PTX, paclitaxel.





**Figure S5. Paclitaxel elevated the expression of acetylated α-tubulin upon NLRP3 inflammasome activation in BMDMs. (A)** Cells were treated in **Fig. 1D**. **(B)** Cells were treated in **Fig. 1G**. Acetylated α-tubulin and total α-tubulin expression levels were determined by Western blotting. PTX, paclitaxel.





**Figure S6 Paclitaxel promoted the levels of acetylated α-tubulin and Nigericin-induced inflammasome activation in THP-1 cells. (A, B)** Human THP-1 cells were differentiated by incubation with PMA (500 nM) for 16 h before being primed with LPS (1 μg/ml) for 4 h, and then pre-treated with graded concentrations of paclitaxel for 1 h. **(A)** Western blotting was used to assess the levels of indicated proteins in cell lysates. Actin was used as the loading control. Histograms showing the quantification of acetylated α-tubulin relative to total α-tubulin are shown in **(B)**. Data were analyzed using the non-parametric Mann–Whitney U test, which are shown as mean ± SD (*n* = 3). **(C)** THP-1 macrophages were pre-treated with series concentrations of paclitaxel for 1 h followed by stimulation with nigericin (20 μM) for 2 h. The levels of soluble IL-1β in culture supernatants were analyzed by cytometric bead array (CBA) assay. Data were analyzed using the One-way ANOVA followed by Turkey post hoc test, which are shown as mean ± SD (*n* = 5). ***P* < 0.01; ****P* < 0.001; PTX, paclitaxel; Nig, nigericin.


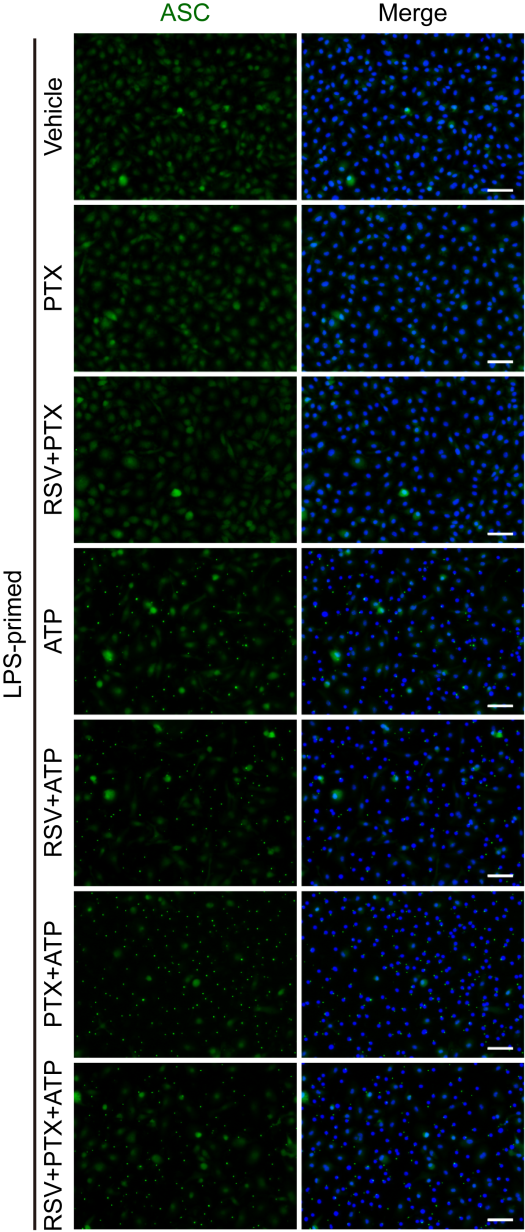


**Figure S7. Resveratrol suppressed ATP-induced ASC speck formation in BMDMs.** LPS-primed BMDMs were pre-treated with resveratrol (5 μM) for 30 min before paclitaxel (100 nM) for 1 h, followed by incubation with ATP (2 mM) for 30 min without LPS. Representative immunofluorescence images showing ASC (green) subcellular distribution. Nuclei (blue) were revealed by Hoechst 33342. The images for ASC and nuclei were captured, respectively, and merged together. Scale bars, 50 μm. PTX, paclitaxel; RSV, resveratrol.





**Figure S8. Epothilone B induces α-tubulin acetylation in BMDMs.** BMDMs were first primed with LPS (500 ng/ml) for 4 h, and then treated with graded concentrations of epothilone B for 1 h. **(A)** Western blotting was used to assess the levels of indicated proteins in cell lysates. Actin was used as the loading control. Histograms showing the quantification of acetylated α-tubulin relative to total α-tubulin are shown in **(B)**, respectively. Data were analyzed using the unpaired Student’s t-test, which are shown as mean ± SD (*n* = 3). ****P* < 0.001. EpoB, Epothilone B.
